# Beyond viral suppression of HIV – the new quality of life frontier

**DOI:** 10.1186/s12916-016-0640-4

**Published:** 2016-06-22

**Authors:** Jeffrey V. Lazarus, Kelly Safreed-Harmon, Simon E. Barton, Dominique Costagliola, Nikos Dedes, Julia del Amo Valero, Jose M. Gatell, Ricardo Baptista-Leite, Luís Mendão, Kholoud Porter, Stefano Vella, Jürgen Kurt Rockstroh

**Affiliations:** ISGlobal, Hospital Clinic, University of Barcelona, Barcelona, Spain; Centre for Health and Infectious Disease Research (CHIP), Rigshospitalet, University of Copenhagen, Copenhagen, Denmark; Imperial College, London, UK; Sorbonne Universités, INSERM, UPMC Univ Paris 06, Institut Pierre Louis d’épidémiologie et de Santé Publique, F75013 Paris, France; European AIDS Treatment Group, Brussels, Belgium; Instituto de Salud Carlos III, Madrid, Spain; Hospital Clínic-IDIBAPS, University of Barcelona, Barcelona, Spain; Universidade Católica Portuguesa, Lisbon, Portugal; Faculty of Health, Medicine and Life Sciences, Maastricht University, Maastricht, The Netherlands; University College London, London, UK; Global Health, Istituto Superiore di Sanità, Rome, Italy; Department of Medicine I, University Hospital Bonn, Bonn, Germany

**Keywords:** AIDS, HIV, Health policy, Health systems

## Abstract

**Background:**

In 2016, the World Health Organization (WHO) adopted a new Global Health Sector Strategy on HIV for 2016–2021. It establishes 15 ambitious targets, including the ‘90-90-90’ target calling on health systems to reduce under-diagnosis of HIV, treat a greater number of those diagnosed, and ensure that those being treated achieve viral suppression.

**Discussion:**

The WHO strategy calls for person-centered chronic care for people living with HIV (PLHIV), implicitly acknowledging that viral suppression is not the ultimate goal of treatment. However, it stops short of providing an explicit target for health-related quality of life. It thus fails to take into account the needs of PLHIV who have achieved viral suppression but still must contend with other intense challenges such as serious non-communicable diseases, depression, anxiety, financial stress, and experiences of or apprehension about HIV-related discrimination. We propose adding a ‘fourth 90’ to the testing and treatment target: ensure that 90 % of people with viral load suppression have good health-related quality of life. The new target would expand the continuum-of-services paradigm beyond the existing endpoint of viral suppression. Good health-related quality of life for PLHIV entails attention to two domains: comorbidities and self-perceived quality of life.

**Conclusions:**

Health systems everywhere need to become more integrated and more people-centered to successfully meet the needs of virally suppressed PLHIV. By doing so, these systems can better meet the needs of all of their constituents – regardless of HIV status – in an era when many populations worldwide are living much longer with multiple comorbidities.

## Introduction

In May 2016, in its 69th session, the World Health Assembly approved a new Global Health Sector Strategy on HIV for 2016–2021 [1]. The goal of the strategy is nothing less than “*to end the AIDS epidemic as a public health threat by 2030*” – an incredible advance from some 15 years ago, when the world set out to put three million people on antiretroviral therapy by the end of 2005.

The strategy, formulated by the World Health Organization (WHO), establishes 15 ambitious global targets that are to be achieved by 2020 (Box 1). These include reducing global HIV-related deaths to below 500,000 annually and fully eliminating mother-to-child transmission of HIV. Further, the importance of continuing the quest for a cure for HIV is acknowledged with an ‘innovation’ target that calls on stakeholders to “*increase research into and development of HIV-related vaccines and medicines*” [1].

Regarding HIV testing and treatment, the strategy puts forth the ‘90-90-90’ target championed by UNAIDS [2]. This target reflects key points across the continuum of HIV services, in keeping with the concept that health systems simultaneously need to reduce under-diagnosis of HIV, treat a greater number of those diagnosed, and ensure that those being treated achieve viral suppression. While reaching the ‘90-90-90’ target would deliver tremendous benefits to individuals already infected with HIV, this target is also important from a public health perspective since achieving such a large increase in the number of virally suppressed people would greatly reduce onward transmission of the disease.

However, what about the millions of people already living with HIV? What next following viral suppression?

The WHO strategy alludes to ‘what next’ by defining its vision as: “*zero new HIV infections, zero HIV-related deaths and zero HIV-related discrimination in a world where people living with HIV are able to live long and healthy lives*” [1]. Furthermore, the strategy’s central goal, fully articulated, is “*to end the AIDS epidemic as a public health threat by 2030, within the context of ensuring healthy lives and promoting well-being for all at all ages*” [1].

## A ‘fourth 90’

**Fig. 1:**
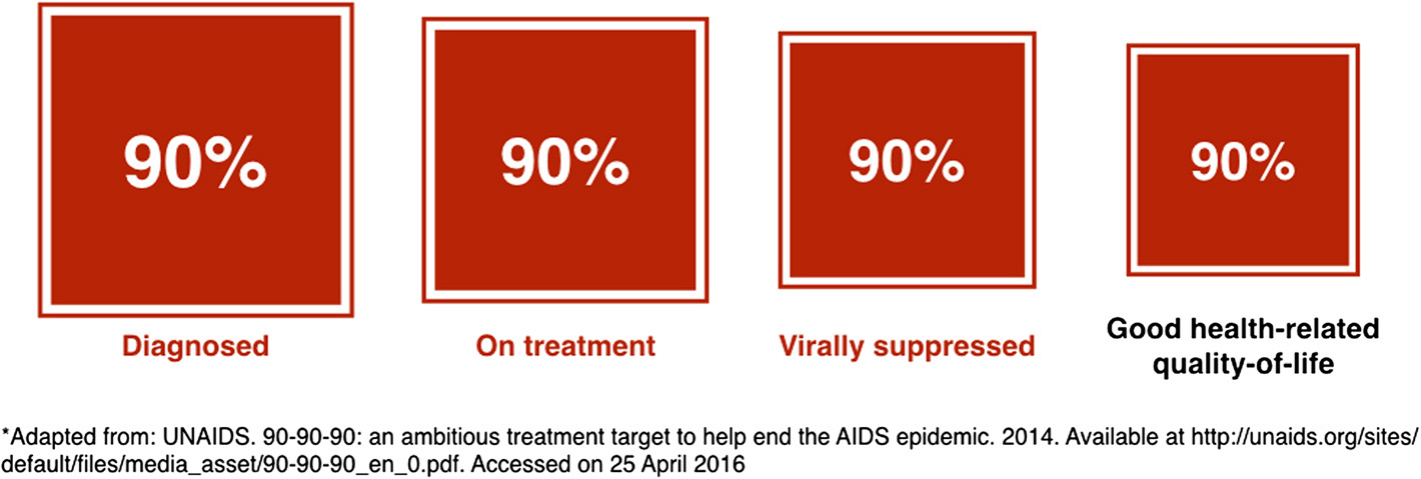
The ‘fourth 90’: proposed revision to the UNAIDS 90-90-90 targets*

This broad framing of the undertaking represents a welcome change from the more simplistic response to HIV in the earlier years of the epidemic. However, the strategy does not provide targets for the concept of ensuring healthy lives and promoting well-being. Thus, we propose adding a ‘fourth 90’ to the testing and treatment target: ensure that 90 % of people with viral load suppression have good health-related quality of life (Fig. 1)*.* We further suggest that good health-related quality of life for PLHIV entails attention to two domains – comorbidities and self-perceived quality of life.

A large body of research has raised questions about whether PLHIV, as a whole, are at elevated risk for non-HIV-specific diseases such as kidney disease, cardiovascular disease and various cancers. A careful parsing of the data on comorbidities in PLHIV indicates that a higher incidence of some of these diseases is concentrated in two subsets of people: those who began taking antiretroviral therapy during the earliest phase of the treatment era and those who have experienced more severe immunosuppression, which is a common consequence of late diagnosis [3–6]. Nonetheless, many questions remain about how to proactively address comorbidities in PLHIV, including virally suppressed PLHIV. As more people taking antiretroviral therapy live into their fifties, sixties and beyond, healthcare providers will increasingly be confronted with scenarios in which multiple diseases need to be managed simultaneously and multiple types of pharmaceutical interventions need to be coordinated.

As for self-perceived quality of life, virally suppressed PLHIV have reported high levels of symptoms such as fatigue and energy loss, insomnia, sadness and depression, sexual dysfunction and changes in body appearance [7]. A cross-sectional study comparing more than 3000 PLHIV with more than 7000 members of the general population in the United Kingdom reported that health-related quality of life scores were significantly lower among PLHIV, 75 % of whom were virally suppressed, and that scores remained significantly lower even when the analysis was restricted to virally suppressed PLHIV [8].

Addressing these and other related concerns is essential in order to ensure that the vision of well-being put forth by the WHO strategy becomes a reality. Indeed, by calling for person-centered chronic care for PLHIV, the strategy acknowledges that viral suppression is not the ultimate goal of treatment – that, apart from reducing HIV incidence and mortality, treatment needs to encompass attention to a comprehensive set of issues, including non-communicable diseases (NCDs), mental health disorders, pain management and palliative care. Further, the strategy clearly recognizes stigma and discrimination as being detrimental to the well-being of PLHIV [1].

**Fig. 2:**
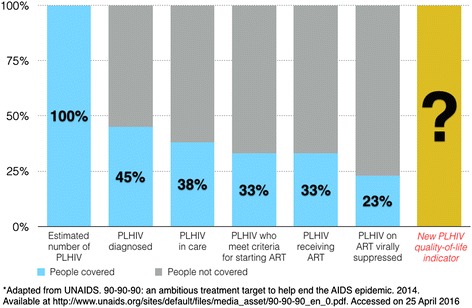
Expanding the HIV continuum of care cascade: an example using data from Vietnam*

**Fig. 3:**
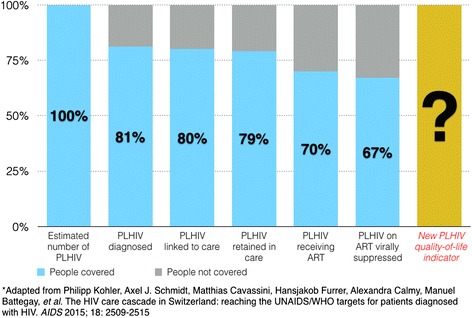
Expanding the HIV continuum of care cascade: an example using data from Switzerland*

This is all well and good – and some elements of the WHO strategy might even be considered visionary in terms of how they seek to address the needs of PLHIV beyond viral suppression. Nevertheless, two issues have not been addressed. One is an explicit target for health-related quality of life – our proposed ‘fourth 90’. The other is policy and operational guidance for how to achieve the ‘fourth 90’. This shortcoming does not reflect on WHO, but rather highlights the need for the global public health community as a whole to develop a new paradigm for ‘*beyond viral suppression*’. More precisely, we need to reconsider the scope of the continuum-of-services paradigm, which has been an enormously useful approach to indicate where efforts should be concentrated in order to prevent fall-off along the continuum in specific national and sub-national contexts. It is now time to add health-related quality of life of PLHIV to the continuum (Figs. 2 and 3).

We thus call on countries that are already well-positioned to fully or partially achieve the UNAIDS 90-90-90 target to lead the way in attending to this matter, and we ask their national governments to formally commit to the ‘fourth 90’. This entails establishing a health-related quality of life target on par with other overarching national HIV testing and treatment targets, as well as developing strategies that will guide health systems in their pursuit of the new target and metrics for tracking their progress.

At first consideration, our proposal may seem relevant only to those with the luxury of addressing HIV– or, for that matter, living with HIV and enjoying unhindered access to health services – in wealthy countries. After all, if less than half of PLHIV in some severely resource-limited countries even have access to antiretroviral therapy, why should the global community worry about the needs of the virally suppressed populations in high-income countries? We can think of multiple reasons.

First, controlling HIV infection does not necessarily eliminate other enormous challenges associated with this disease. Serious NCDs, depression, anxiety, financial stress, fear of transmitting HIV to others, uncertainty about starting a family, and experiences of or apprehension about HIV-related discrimination – these and a host of other issues prevent many PLHIV from relegating their HIV infection to the periphery of daily life. In the coming years, healthcare models developed in high-income countries in response to these issues will directly inform how health systems in low- and middle-income countries seek to address the needs of large cohorts of PLHIV who are taking antiretroviral drugs on a lifelong basis. Introducing and pursuing a ‘fourth 90’ target to address the challenges facing virally suppressed PLHIV in high-income countries will thus set an important precedent for other countries to follow.

Another consideration is late diagnosis of HIV, which remains a major problem in countries at all income levels [9]. Research suggests that, even after achieving viral suppression, people who commenced antiretroviral therapy with severely weakened immune systems are vulnerable to a range of HIV- and non-HIV-specific diseases [10–12]. The ideal, of course, would be to eliminate late diagnosis. However, many health systems worldwide continue to grapple with the question of how to incentivize HIV testing even enough to achieve the ‘first 90’ target of diagnosing 90 % of HIV-positive people [13]. While work on this front continues, increasing our understanding of the health needs of virally suppressed PLHIV, including those diagnosed late, will yield clinical and operational guidance that will be of immense value in virtually all healthcare settings worldwide.

Our final reason for urging suitably positioned countries to rise to the challenge of exploring what is needed for PLHIV “*beyond viral suppression*” relates to the changing dynamics of health systems. In recent years, health systems experts have begun calling for health services and other health system elements to be centered on the needs of individuals and communities, which would be a departure from the disease-specific orientation that currently characterizes many aspects of health systems worldwide. However, there is not yet a clear consensus about what ‘people-centered health systems’ should look like in practice [14]. This issue has emerged at a time when health systems in some low- and middle-income countries are being severely strained by the rising incidence in various NCDs [15].

## Conclusions

We believe that health systems everywhere need to become more integrated and people-centered to successfully meet the needs of virally suppressed PLHIV. Importantly, by doing so, these systems can better meet the needs of all of their constituents, regardless of HIV status, in an era when many populations worldwide are living much longer with multiple comorbidities. It is imperative for the global community to approach health-related well-being holistically from this perspective. We therefore challenge our colleagues working on HIV worldwide to take up the ‘fourth 90’ – ensure that 90 % of people with viral load suppression have good health-related quality of life – as a central guiding objective in the next stage of the march to end HIV.

## Box 1. Draft global health sector strategy on HIV, 2016–2021: global targets for 2020

HIV-related deaths:

- Reduce global HIV-related deaths to below 500,000
- Reduce tuberculosis deaths among people living with HIV by 75 %
- Reduce hepatitis B and C deaths among people co-infected with HIV by 10 %, in line with mortality targets for all people with chronic hepatitis B and C infection

Testing and treatment:

- Ensure that 90 % of people living with HIV know their HIV status
- Ensure that 90 % of people diagnosed with HIV receive antiretroviral therapy
- Ensure that 90 % of people living with HIV, and who are on treatment, achieve viral load suppression

Prevention:

- Reduce new HIV infections to below 500,000
- Zero new infections among infants

Discrimination:

- Zero HIV-related discriminatory laws, regulations and policies, and zero HIV-related discrimination in all settings, especially health settings
- Ensure that 90 % of people living with HIV and key populations report no discrimination in the health sector

Financial sustainability:

- Ensure financial risk protection for 90 % of all people living with HIV
- Ensure domestic investments in upper middle-income countries of 95 % of national AIDS resource needs and annual international HIV investment of US$ 12,700 million in lower middle-income countries;
- Ensure all countries have integrated essential HIV services into national health financing arrangements

Innovation:

- Increase research into and development of HIV-related vaccines and medicines for use in treatment and prevention
- Provision of access by 90 % of countries to integrated health services covering HIV, tuberculosis, hepatitis B and C, reproductive health and sexually transmitted infections

*Source:* [1]
